# Evaluating the efficacy and safety of human anti-SARS-CoV-2 convalescent plasma in severely ill adults with COVID-19: A structured summary of a study protocol for a randomized controlled trial

**DOI:** 10.1186/s13063-020-04422-y

**Published:** 2020-06-08

**Authors:** Christina M. Eckhardt, Matthew J. Cummings, Kartik N. Rajagopalan, Sarah Borden, Zachary C. Bitan, Allison Wolf, Alex Kantor, Thomas Briese, Benjamin J. Meyer, Samuel D. Jacobson, Dawn Scotto, Nischay Mishra, Neena M. Philip, Brie A. Stotler, Joseph Schwartz, Beth Shaz, Steven L. Spitalnik, Andrew Eisenberger, Eldad A. Hod, Jessica Justman, Ken Cheung, W. Ian Lipkin, Max R. O’Donnell

**Affiliations:** Columbia University Medical Center, New York, USA

**Keywords:** COVID-19, SARS-CoV-2, Respiratory Failure, Randomized controlled trial, Protocol, Convalescent Plasma, Anti-SARS-CoV-2 plasma

## Abstract

**Objectives:**

The aim of this study is to evaluate the efficacy and safety of human anti-SARS-CoV-2 convalescent plasma in hospitalized adults with severe SARS-CoV-2 infection.

**Trial Design:**

This is a prospective, single-center, phase 2, randomized, controlled trial that is blinded to participants and clinical outcome assessor.

**Participants:**

Eligible participants include adults (≥ 18 years) with evidence of SARS-CoV-2 infection by PCR test of nasopharyngeal or oropharyngeal swab within 14 days of randomization, evidence of infiltrates on chest radiography, peripheral capillary oxygen saturation (SpO2) ≤ 94% on room air, and/or need for supplemental oxygen, non-invasive mechanical ventilation, or invasive mechanical ventilation, who are willing and able to provide written informed consent prior to performing study procedures or who have a legally authorized representative available to do so. Exclusion criteria include participation in another clinical trial of anti-viral agent(s)* for coronavirus disease-2019 (COVID-19), receipt of any anti-viral agent(s)* with possible activity against SARS-CoV-2 <24 hours prior to plasma infusion, mechanical ventilation (including extracorporeal membrane oxygenation [ECMO]) for ≥ 5 days, severe multi-organ failure, history of allergic reactions to transfused blood products per NHSN/CDC criteria, known IgA deficiency, and pregnancy. Included participants will be hospitalized at the time of randomization and plasma infusion.

*Use of remdesivir as treatment for COVID-19 is permitted.

The study will be undertaken at Columbia University Irving Medical Center in New York, USA.

**Intervention and comparator:**

The investigational treatment is anti-SARS-CoV-2 human convalescent plasma. To procure the investigational treatment, volunteers who recovered from COVID-19 will undergo testing to confirm the presence of anti-SARS-CoV-2 antibody to the spike trimer at a 1:400 dilution. Donors will also be screened for transfusion-transmitted infections (e.g. HIV, HBV, HCV, WNV, HTLV-I/II, T. *cruzi,* ZIKV). If donors have experienced COVID-19 symptoms within 28 days, they will be screened with a nasopharyngeal swab to confirm they are SARS-CoV-2 PCR-negative. Plasma will be collected using standard apheresis technology by the New York Blood Center. Study participants will be randomized in a 2:1 ratio to receive one unit (200 – 250 mL) of anti-SARS-CoV-2 plasma versus one unit (200 – 250 mL) of the earliest available control plasma. The control plasma cannot be tested for presence of anti-SARS-CoV-2 antibody prior to the transfusion, but will be tested for anti- SARS-CoV-2 antibody after the transfusion to allow for a retrospective per-protocol analysis.

**Main outcomes:**

The primary endpoint is time to clinical improvement. This is defined as time from randomization to either discharge from the hospital or improvement by one point on the following seven-point ordinal scale, whichever occurs first.

1. Not hospitalized with resumption of normal activities

2. Not hospitalized, but unable to resume normal activities

3. Hospitalized, not requiring supplemental oxygen

4. Hospitalized, requiring supplemental oxygen

5. Hospitalized, requiring high-flow oxygen therapy or non-invasive mechanical ventilation

6. Hospitalized, requiring ECMO, invasive mechanical ventilation, or both

7. Death

This scale, designed to assess clinical status over time, was based on that recommended by the World Health Organization for use in determining efficacy end-points in clinical trials in hospitalized patients with COVID-19. A recent clinical trial evaluating the efficacy and safety of lopinavir- ritonavir for patients hospitalized with severe COVID-19 used a similar ordinal scale, as have recent clinical trials of novel therapeutics for severe influenza, including a post-hoc analysis of a trial evaluating immune plasma.

The primary safety endpoints are cumulative incidence of grade 3 and 4 adverse events and cumulative incidence of serious adverse events during the study period.

**Randomization:**

Study participants will be randomized in a 2:1 ratio to receive anti-SARS-CoV-2 plasma versus control plasma using a web-based randomization platform. Treatment assignments will be generated using randomly permuted blocks of different sizes to minimize imbalance while also minimizing predictability.

**Blinding (masking):**

The study participants and the clinicians who will evaluate post-treatment outcomes will be blinded to group assignment. The blood bank and the clinical research team will not be blinded to group assignment.

**Numbers to be randomized (sample size):**

We plan to enroll 129 participants, with 86 in the anti-SARS-CoV-2 arm, and 43 in the control arm. Among the participants, we expect ~70% or n = 72 will achieve clinical improvement. This will yield an 80% power for a one-sided Wald test at 0.15 level of significance under the proportional hazards model with a hazard ratio of 1.5.

**Trial Status:**

Protocol AAAS9924, Version 17APR2020, 4/17/2020

Start of recruitment: April 20, 2020

Recruitment is ongoing.

**Trial registration:**

ClinicalTrials.gov: NCT04359810

Date of trial registration: April 24, 2020

Retrospectively registered

**Full protocol:**

The full protocol is attached as an additional file, accessible from the Trials website (Additional file 1). In the interest of expediting dissemination of this material, the familiar formatting has been eliminated; this Letter serves as a summary of the key elements of the full protocol.

## Supplementary information

**Additional file 1.** Full Study Protocol.

## Data Availability

Only authorized personnel directly involved with the study will have access to the de-identified, coded, computerized database. Participant’s records will be available to study investigators, the FDA, the NIH, the manufacturer of the study product, and the IRB.

## Abbreviations

CDC: Centers for Disease Control and Prevention
HBV: Hepatitis B Virus
HCV: Hepatitis C Virus
HIV: Human Immunodeficiency Virus
HTLV-I/II: Human T-cell Lymphotropic Virus Type I/II
IgA: Immunoglobulin A
NHSN: National Healthcare Safety Network
PCR: Polymerase chain reaction
SARS-CoV-2: Severe acute respiratory syndrome coronavirus 2
T. cruzi: Trypanosoma cruzi
WNV: West Nile virus
ZIKV: Zika virus

