# Correction to: Evaluating the efficacy and safety of human anti-SARS-CoV-2 convalescent plasma in severely ill adults with COVID-19: A structured summary of a study protocol for a randomized controlled trial

**DOI:** 10.1186/s13063-020-04877-z

**Published:** 2020-11-17

**Authors:** Christina M. Eckhardt, Matthew J. Cummings, Kartik N. Rajagopalan, Sarah Borden, Zachary C. Bitan, Allison Wolf, Alex Kantor, Thomas Briese, Benjamin J. Meyer, Samuel D. Jacobson, Dawn Scotto, Nischay Mishra, Neena M. Philip, Brie A. Stotler, Joseph Schwartz, Beth Shaz, Steven L. Spitalnik, Andrew Eisenberger, Eldad A. Hod Jessica Justman, Ken Cheung, W. Ian Lipkin, Max R. O’Donnell

**Affiliations:** Columbia University Medical Center, New York, USA

**Correction to: Trials 21, 499 (2020)**

**https://doi.org/10.1186/s13063-020-04422-y**

Following publication of the original article [1], we were notified that in August 2020, the trial transitioned from a single-center to a multi-center trial and began enrolling participants at three additional clinical sites in Brazil: Instituto Nacional de Infectologia Evandro Chagas, Hospital Federal dos Servidores do Estado, and Hospital Geral de Nova lguaçu. The trial received approval from the appropriate ethical committees at each site. With the expansion, the primary endpoint of the study was also amended. The original primary endpoint was time-to-clinical improvement from enrollment up to study day 28. However, after observing that patients’ clinical statuses can fluctuate, it became clear that the initial primary endpoint would not reflect instances when patients’ clinical outcomes worsen after a period of initial improvement. Thus, the primary endpoint of the study was amended to be the day 28 severity outcome on a seven-point ordinal scale:

1. Not hospitalized with resumption of normal activities.
2. Not hospitalized, but unable to resume normal activities.
3. Hospitalized, not requiring supplemental oxygen.
4. Hospitalized, requiring supplemental oxygen.
5. Hospitalized, requiring high-flow oxygen therapy or noninvasive mechanical ventilation.
6. Hospitalized, requiring extracorporeal membrane oxygenation (ECMO), invasive mechanical ventilation, or both.
7. Death.

After the primary endpoint was amended, the sample size was re-calculated. The calculation was based on blinded pooled data of the day 28 outcome and an odds ratio of 1.7 under a proportional odds assumption. With a 2:1 randomization ratio and a total sample size of 219 participants (146 in the convalescent plasma arm versus 73 in the control arm), a one-sided Mann-Whitney test at a level of 15% will have 82% power to detect an odds ratio of 1.7. At the time the study endpoint was amended, we noted that a recent trial of remdesivir yielded an odds ratio of 1.50 with 95% CI 1.18–1.91, which covered our assumed odds ratio [2]. Thus, we planned to enrol a total of 219 participants across four clinical sites.

Given data suggesting a potential signal of benefit of convalescent plasma therapy in specific patient subgroups [3, 4], pre-specified subgroup analyses will be conducted for the following outcomes: day 28 severity outcome, in-hospital and 28-day mortality, and time-to-clinical improvement. The following subgroups will be examined: level of oxygen support at baseline (no supplemental oxygen, supplemental oxygen [including high-flow nasal cannula oxygen], mechanical ventilation or ECMO) and symptom duration at baseline (≤7 days, > 7 days).
