# Supplementary material for: Evaluating the efficacy and safety of human anti-SARS-CoV-2 convalescent plasma in severely ill adults with COVID-19: A structured summary of a study protocol for a randomized controlled trial
Source: Trials. 2020 Jun 8;21:499. doi: 10.1186/s13063-020-04422-y (PMC7276974; doi:10.1186/s13063-020-04422-y)
Supplement: Supplementary file 1 — Additional file 1. Full Study Protocol. [file 13063_2020_4422_MOESM1_ESM.docx]

# PROTOCOL SUMMARY:

**Long title:** A Phase 2, Randomized Clinical Trial to Evaluate the Efficacy and Safety of Human Anti-SARS-CoV-2 Convalescent Plasma in Severely Ill Adults with COVID-19

**Short title**: Population A: Convalescent Plasma in Severe COVID-19

**Clinical Phase**: 2

**IND Sponsor**: Andrew B. Eisenberger, MD

**Principal Investigator**: Max O’Donnell, MD, MPH

**Conducted by:** Columbia University

**Sample Size:** 129

**Study Population**: Subjects aged ≥18 years with severe COVID-19

**Study Duration:** Approximately 1 year.

**Study Design:** Randomized blinded phase 2 trial to assess the efficacy and safety of anti-SARS-CoV-2 plasma among adults with severe COVID-19. A total of 129 eligible subjects will be randomized in a 2:1 ratio to receive either convalescent plasma qualitatively positive for SARS-CoV-2 antibody (anti-SARS-CoV-2 plasma) or non-convalescent fresh frozen plasma (control plasma). The patient and the study clinician assessing the clinical outcome will be blinded to the treatment arm.

The following will be assessed in all subjects:

Safety and efficacy: Day 0 (baseline) to Day 28.

**Study Agent:**

- Anti-SARS-CoV-2 convalescent plasma (1 unit; ~200-250 mL collected by apheresis from a volunteer who recovered from COVID-19 (collection and qualification covered by IRB protocol AAAS9845 (Convalescent plasma donors)) and was found to be a qualitatively positive for SARS-CoV-2 antibody (“anti-SARS-CoV-2 plasma”).
- Control plasma: 1 unit of standard plasma collected prior to December 2019

**Primary Efficacy Objective:** To evaluate the efficacy of treatment with anti-SARS-CoV-2 plasma versus control plasma with respect to time-to-clinical improvement, defined as the time from randomization to either an improvement of one point on a seven-category ordinal scale or alive discharge from the hospital, whichever comes first followed through Day 28.

**Primary Endpoint:** Time-to-clinical improvement, defined as the time from randomization to either an improvement of one point on a seven-category ordinal scale or alive at discharge from the hospital, whichever comes first. The seven-category ordinal scale consists of the following categories:

1. Not hospitalized with resumption of normal activities
2. Not hospitalized, but unable to resume normal activities
3. Hospitalized, not requiring supplemental oxygen
4. Hospitalized, requiring supplemental oxygen
5. Hospitalized, requiring high-flow oxygen therapy or noninvasive mechanical ventilation
6. Hospitalized, requiring extracorporeal membrane oxygenation (ECMO), invasive mechanical ventilation, or both
7. Death

**Primary Safety Objective:** To evaluate the safety and tolerability of treatment with anti- SARS-CoV-2 plasma versus control (control plasma) in adults with severe COVID-19.

**Primary Safety Endpoints:**

1. Cumulative incidence of grade 3 and 4 adverse events during the study period
2. Cumulative incidence of serious adverse events during the study period

**Secondary Objectives:**

1. To compare the proportion and duration of SARS-CoV-2 PCR positivity (RT PCR) between the recipients of the anti-SARS-CoV-2 plasma and control plasma at days 0, 7, and 14.
2. To compare the levels of SARS-CoV-2 RNA between recipients of the anti-SARS-CoV-2 plasma and control plasma at days 0, 7, and 14.
3. To compare duration of need for supplemental oxygen and/or mechanical ventilation between recipients of the anti-SARS-CoV-2 plasma and control plasma.
4. To compare duration of hospitalization between recipients of the anti-SARS-CoV-2 plasma and control plasma.
5. To compare in-hospital and 28-day mortality between recipients of the anti-SARS-CoV-2 plasma and control plasma.
6. To assess for genetic and transcriptomic differences at Day 0 (genomic) and Day 0,7,14 (transcriptomic) between the recipients of the anti-SARS-CoV-2 plasma and control plasma.

Table of Contents

[PROTOCOL SUMMARY: 1](#_Toc37942570)

[1. STUDY POPULATION 5](#_Toc37942571)

[2. LIST OF ABBREVIATIONS 6](#_Toc37942574)

[3. BACKGROUND AND SCIENTIFIC RATIONALE 7](#_Toc37942575)

[1. Experience with the use of convalescent plasma against coronavirus diseases 8](#_Toc37942576)

[2. Overview of known potential risks 8](#_Toc37942577)

[3. Known potential benefits 9](#_Toc37942578)

[4. INVESTIGATIONAL PLAN 11](#_Toc37942579)

[1. Study Objectives 11](#_Toc37942580)

[2. Definitions 11](#_Toc37942583)

[3. Study population 12](#_Toc37942584)

[4. Study product considerations 15](#_Toc37942596)

[5. STATISTICAL CONSIDERATIONS AND DATA MANAGEMENT 17](#_Toc37942634)

[5.1. Design Overview 17](#_Toc37942635)

[5.2. Statistical Analysis 18](#_Toc37942636)

[1. Primary Analysis, go/no-go decision, and power consideration 18](#_Toc37942637)

[2. Secondary Analysis 19](#_Toc37942638)

[3. Safety Analyses 19](#_Toc37942639)

[4. Missing data and non-compliance 19](#_Toc37942640)

[5.3. Go/no-go Decision and Power Consideration 19](#_Toc37942658)

[6. STUDY PROCEDURES 20](#_Toc37942659)

[Day -1 to 0 20](#_Toc37942660)

[Day 0 Baseline 20](#_Toc37942661)

[Day 0 Infusion 21](#_Toc37942661)

[Day 1 ± 1 21](#_Toc37942662)

[Day 3 ± 2 21](#_Toc37942663)

[Day 7 ± 2 21](#_Toc37942664)

[Day 14 ± 2 22](#_Toc37942665)

[Day 28 ± 3 22](#_Toc37942666)

[7. EFFICACY, VIROLOGIC AND PK MEASURES 22](#_Toc37942667)

[8. RISKS AND BENEFITS 23](#_Toc37942668)

[8.1. Potential benefits of treatment 23](#_Toc37942669)

[8.2. Potential benefits of clinical monitoring and virologic testing 23](#_Toc37942670)

[8.3. Potential risks of study procedures 23](#_Toc37942671)

[8.4. Potential risks of genetic testing 23](#_Toc37942672)

[8.5. Alternatives 24](#_Toc37942673)

[8.6. Safety monitoring 24](#_Toc37942674)

[8.7. Adverse event reporting 24](#_Toc37942675)

[9. SAFETY OVERSIGHT 29](#_Toc37942676)

[9.1. Monitoring Plan 29](#_Toc37942677)

[9.2. Study monitoring 29](#_Toc37942678)

[9.3. Halting Criteria for the Study 30](#_Toc37942679)

[10. ETHICS/PROTECTION OF HUMAN SUBJECTS 31](#_Toc37942680)

[10.1. Ethical Standard 31](#_Toc37942681)

[10.2. Institutional Review Board 32](#_Toc37942682)

[10.3. Informed Consent Process 32](#_Toc37942684)

[10.4. Subject Confidentiality 32](#_Toc37942685)

[10.5. Future Use of Stored Specimens 33](#_Toc37942686)

[10.6. Data management and monitoring 33](#_Toc37942687)

[11. REFERENCES 35](#_Toc37942694)

# STUDY POPULATION

### Inclusion Criteria for Enrollment

1. Willing and able to provide written informed consent prior to performing study procedures or have a legally authorized representative available to do so.
2. Age ≥18 years.
3. Evidence of SARS-CoV-2 infection by PCR test of nasopharyngeal swab or oropharyngeal swab/tracheal aspirate sample within 14 days of randomization.
4. Peripheral capillary oxygen saturation (SpO2) ≤ 94% on room air or requiring supplemental oxygen, non-invasive or invasive mechanical ventilation at screening.
5. Evidence of infiltrates on chest radiography.
6. Females of childbearing age and males must be willing to practice an effective contraceptive method or remain abstinent during the study period.

### Exclusion Criteria

1. Participation in another clinical trial of anti-viral agent(s)* for COVID-19.
2. Receipt of any anti-viral agent(s)* with possible activity against SARS-CoV-2 <24 hours prior to study drug administration.
3. Mechanically ventilated (including veno-venous (VV)-ECMO) ≥ 5 days.
4. Severe multi-organ failure.
5. History of prior reactions to transfusion blood products meeting definitive case definition criteria, at least severe severity, and probable or definite imputability per National Healthcare Safety Network (NHSN)/Centers for Disease Control and Prevention (CDC) criteria^[[1]](#endnote-1)^.
6. Known IgA deficiency.
7. Females who are pregnant or breastfeeding.

*Use of Remdesivir as treatment for COVID-19 is permitted.

# LIST OF ABBREVIATIONS

ADR: Adverse Drug Reaction

ADE: Antibody-mediated enhancement of infection

AE: Adverse Event/Adverse Experience

CDC: United States Centers for Disease Control and Prevention

CFR: Code of Federal Regulations

CLIA: Clinical Laboratory Improvement Amendment of 1988

COI: Conflict of Interest

COVID-19: Coronavirus Disease

CRF: Case Report Form

DCC: Data Coordinating Center

DMC: Data Management Center

DSMB: Data and Safety Monitoring Board

EUA: Emergency Use Authorization

FDA: Food and Drug Administration

GCP: Good Clinical Practice

HBV: Hepatitis B virus

HCIP: Human Coronavirus Immune Plasma

HCV: Hepatitis C virus

HIV: Human immunodeficiency virus

HTLV: Human T-cell lymphotropic virus

IB: Investigator’s Brochure

ICF: Informed Consent Form

ICH: International Conference on Harmonization

ICU: Intensive Care Unit

IND: Investigational New Drug Application

IRB: Institutional review board

ISBT: International Society of Blood Transfusion

MERS: Middle East Respiratory Syndrome

RT-PCR: Reverse Transcriptase Polymerase chain reaction

PK: Pharmacokinetic

PPE: Personal Protective Equipment

SAE: Serious adverse event

SARS: Severe Acute Respiratory Syndrome

SARS-CoV-2: Severe Acute Respiratory Syndrome Coronavirus 2

TACO: Transfusion-associated circulatory overload

TRALI: Transfusion-related acute lung injury

UP: Unanticipated Problem

# BACKGROUND AND SCIENTIFIC RATIONALE

There are currently no proven treatment options for coronavirus disease (COVID-19), which is caused by Severe Acute Respiratory Syndrome Coronavirus 2 (SARS-CoV-2). Human convalescent plasma has been successfully used for treatment of other severe coronavirus infections and thus may provide an option for treatment of COVID-19 and could be rapidly available from people who have recovered from disease and can donate plasma.

Passive antibody therapy involves the administration of antibodies against a given infectious agent to a susceptible or ill individual for the purpose of preventing or treating an infectious disease caused by that agent. Experience from prior outbreaks with other coronaviruses, such as SARS-CoV-1, shows that convalescent plasma contains neutralizing antibodies to the relevant virus (Zhang et al., 2005). In the case of SARS-CoV-2, the anticipated mechanism of action by which passive antibody therapy would mediate protection is viral neutralization. However, other mechanisms may be possible, such as antibody dependent cellular cytotoxicity and/or phagocytosis. Convalescent serum was also used in the 2013 African Ebola epidemic. A small 73 non-randomized study in Sierra Leone revealed a significant increase in survival for those 74 treated with convalescent whole blood relative to those who received standard treatment (Sahr et al., 2017). The only antibody type that is currently available for immediate use against SARS-CoV-2 is that found in human convalescent plasma. As more individuals contract COVID-19 and recover, the number of potential donors will continue to increase.

When used for therapy, antibody is most effective when administered shortly after the onset of symptoms. The reason for temporal variation in efficacy is not well understood but could reflect that passive antibody works by neutralizing the initial inoculum, which is likely to be much smaller than that of established disease. Another explanation is that antibody works by modifying the inflammatory response, which is also easier during the initial immune response, which may be asymptomatic (Casadevall & Pirofski, 2003). As an example, passive antibody therapy for pneumococcal pneumonia was most effective when administered shortly after the onset of symptoms and there was no benefit if antibody administration was delayed past the third day of disease (Casadevall & Scharff, 1994).

For passive antibody therapy to be effective, a sufficient amount of antibody must be administered. When given to a susceptible person, this antibody will circulate in the blood, reach tissues and provide protection against infection. Depending on the antibody amount and composition, the protection conferred by the transferred immunoglobulin can last from weeks to months.

## Experience with the use of convalescent plasma against coronavirus diseases

In the 21st century, there were two other epidemics with coronaviruses that were associated with high mortality, SARS1 in 2003 and MERS in 2012. In both outbreaks, the high mortality and absence of effective therapies led to the use of convalescent plasma. The largest study involved the treatment of 80 patients in Hong Kong with SARS (Cheng et al., 2005). Patients treated before day 14 had improved prognosis defined by discharge from hospital before day 22, consistent with the observation that earlier administration is more likely to be effective. In addition, those who were PCR positive and seronegative for coronavirus at the time of therapy had improved prognosis. There is also some anecdotal information on the use of convalescent plasma in seriously ill individuals. Three patients with SARS in Taiwan were treated with 500 ml of convalescent plasma, resulting in a reduction in plasma virus titer and each survived (Yeh et al., 2005). Three patients with MERS in South Korea were treated with convalescent plasma, but only two of the recipients had neutralizing antibody in their plasma (Ko et al., 2018). The latter study highlights a challenge in using convalescent plasma, namely, that some who recover from viral disease may not have high titers of neutralizing antibody (Arabi et al., 2016). Consistent with this point, an analysis of 99 samples of convalescent sera from patients with MERS showed that 87 had neutralizing antibody with a geometric mean titer of 1:61. This suggests that antibody declines with time and/or that few patients make high titer responses.

It is also possible that other types of non-neutralizing antibodies are made that contribute to protection and recovery as described for other viral diseases (Gunn et al., 2018; van Erp, Luytjes, Ferwerda, & van Kasteren, 2019). There are reports that convalescent plasma was used for therapy of patients with COVID-19 in China during the current outbreak (Xinhua, 2020). Although few details are available from the Chinese experience and published studies involved small numbers of patients, the available information suggests that convalescent plasma administration reduces viral load and was safe.

## Overview of known potential risks

Historical and current anecdotal data on use of convalescent plasma suggest it is safe in coronavirus infection. Therefore, the large number of exposed healthcare workers, public servants and first responders, in combination with high morbidity and mortality in severe COVID-19, particularly in elderly and vulnerable persons, suggest that the benefits of convalescent plasma outweigh its possible risks in patients with severe illness. However, for all cases where convalescent plasma administration is considered, a risk-benefit assessment must be conducted to assess individual variables.

The theoretical risk involves the phenomenon of antibody-mediated enhancement of infection (ADE). ADE can occur for several viral diseases and involves an enhancement of disease in the presence of certain antibodies. For coronaviruses, several mechanisms for ADE have been described and there is the theoretical concern that antibodies to one type of coronavirus could enhance infection to another viral strain (Wan et al., 2020). It may be possible to predict the risk of ADE of SARS-CoV-2 experimentally, as proposed for MERS. Since the proposed use of convalescent plasma in the COVID-19 epidemic would rely on preparations with high titers of neutralizing antibody against the same virus, SARS2-CoV-2, ADE may be unlikely. The available evidence from the use of convalescent plasma in patients with SARS1 and MERS (Mair-Jenkins et al., 2015), and anecdotal evidence of its use in patients with COVID-19 (Xinhua, 2020), suggest it is safe. Nevertheless, caution and vigilance will be required in for any evidence of enhanced infection.

Another theoretical risk is that antibody administration to those exposed to SARS-CoV-2 may avoid disease but modify the immune response such that those individuals mount attenuated immune responses, which would leave them vulnerable to subsequent re-infection. In this regard, passive antibody administration before vaccination with respiratory syncytial virus was reported to attenuate humoral but not cellular immunity (Crowe, Firestone, & Murphy, 2001). This concern will be investigated as part of this clinical trial by measuring immune responses in those exposed and treated with convalescent plasma to prevent disease. If the concern proved real these individuals could be vaccinated against COVID-19 when a vaccine becomes available.

Passive antibodies are derived from human serum. The antibodies used in this study will be derived from serum obtained from convalescent patients, and will be subjected to testing protocols that are similar to those used by blood banks and transfusion services. However, as is the case with any biological product, there is a very small risk of allergy/anaphylaxis, or passive transfer of potential unknown infectious agents or infections. While most adverse effects are mild and transient including headaches, flushing, fever, chills, fatigue, nausea, diarrhea, transient changes in blood pressure and tachycardia, there is also the risk of transfusion related acute lung injury (TRALI), and transfusion associated circulatory overload (TACO), which could worsen hypoxemia in patients requiring supplemental oxygen or non-invasive or mechanical ventilation. Late adverse events are rare and include acute renal failure and thromboembolic events.

## Known potential benefits

A benefit of convalescent plasma administration is that it can prevent infection and subsequent disease in those who are at high risk for disease following close contacts of patients with COVID-19. This is especially so for those with underlying medical conditions. Many who will qualify for prophylaxis are health care workers and first responders who are critical to maintenance of stability of the healthcare system. Passive antibody administration to prevent disease is already used in clinical practice. For example, patients exposed to hepatitis B and rabies viruses are treated with hepatitis B immune globulin (HBIG) and human rabies immune globulin (RIG), respectively. Botulism Immune Globulin Intravenous (Human) (BIG-IV) is an intravenous preparation for infant botulism. In addition, passive antibody is used for the prevention of severe respiratory syncytial virus (RSV) disease in high-risk infants. Until recently, polyclonal hyperimmune globulin (RSV-IG) prepared from donors selected for having high plasma titers of RSV neutralizing antibody, was used but these preparations have now been replaced by palivizumab, a humanized murine monoclonal antibody.

Another potential benefit is societal: If the frequency with which exposed persons become infected decreases, the risk of further transmission (R naught) might be reduced and the epidemic slowed. Another avenue (not pursued in this protocol) is as a treatment for established infection. Convalescent plasma would be administered to those with clinical disease in an effort to reduce their symptoms and mortality. Based on the historical experience with antibody administration, it can be anticipated that antibody administration would be more effective in preventing disease than in the treatment of established disease. However, potential benefits in patients with known infection include reduced severity of symptoms, reduced duration of hospitalization, reduced likelihood of death due to infection, and increased speed of recovery.

Given that historical and current anecdotal data on use of convalescent plasma suggest it is safe in coronavirus infection, the high mortality of COVID-19, particularly in elderly and vulnerable persons, suggests that the benefits of its use in those at high risk for or with early disease outweigh the risks. However, for all cases where convalescent plasma administration is considered, a risk-benefit assessment must be conducted to assess individual variables.

# INVESTIGATIONAL PLAN

## Study Objectives

### Primary Objectives:

**Primary Efficacy Objective:** To evaluate the efficacy of treatment with anti-SARS-CoV-2 plasma versus control plasma with respect to time-to-clinical improvement, defined as the time from randomization to either an improvement of one point on a seven-category ordinal scale or alive at discharge from the hospital, whichever comes first, followed through Day 28.

**Primary Safety Objective:** To evaluate the safety and tolerability of treatment with anti- SARS-CoV-2 plasma versus control plasma in adults with severe COVID-19.

### Secondary Objectives:

1. To compare the proportion and duration of SARS-CoV-2 PCR positivity (RT PCR) between recipients of the anti-SARS-CoV-2 plasma and control plasma at days 0, 7, and 14.
2. To compare the levels of SARS-CoV-2 RNA between recipients of the anti-SARS-CoV-2 plasma and control plasma at days 0, 7, and 14.
3. To compare duration of need for supplemental oxygen and/or mechanical ventilation between recipients of the anti-SARS-CoV-2 plasma and control plasma.
4. To compare duration of hospitalization between recipients of the anti-SARS-CoV-2 plasma and control plasma.
5. To compare in-hospital and 28-day mortality between recipients of the anti-SARS-CoV-2 plasma and control plasma.
6. To compare genetic differences between recipients of the anti-SARS-CoV-2 plasma and control plasma to explore possible associations with poor outcome.
7. To compare transcriptomic differences between recipients of the anti-SARS-CoV-2 plasma and control plasma at Day 0, 7, and 14 to explore possible associations with poor outcome.

## Definitions

1. Enrolled: From time consented to participate until designated as a screen failure or have either been discontinued from the study or completed it.
2. Randomized: when a study arm is assigned.
3. Screen Failures: signed informed consent, but then determined to be ineligible or withdraws before being randomized.
4. Discontinued: randomized, but then withdrawn by investigator or withdraws consent.
5. Completed: Subjects are considered completed when they are followed through to day 28, if they die before day 28, or are discharged prior to day 14.

## Study population

### Inclusion Criteria for Enrollment

1. Willing and able to provide written informed consent prior to performing study procedures or having a legally authorized representative available to do so.
2. Age ≥18 years.
3. Evidence of SARS-CoV-2 infection by PCR test of nasopharyngeal swab or oropharyngeal swab/tracheal aspirate sample within 14 days of randomization
4. SPO2 ≤ 94% on room air or requiring supplemental oxygen, non-invasive or invasive mechanical ventilation at screening
5. Evidence of infiltrates on chest radiography
6. Females of childbearing age and males must be willing to practice an effective contraceptive method or remain abstinent during the study period.

### Exclusion Criteria for Enrollment

1. Participation in another clinical trial of anti-viral agent(s)* for COVID-19
2. Receipt of any anti-viral agent(s)* with possible activity against SARS-CoV-2 <24 hours prior to plasma infusion
3. Mechanically ventilated (including VV-ECMO) ≥ 5 days
4. Severe multi-organ failure
5. History of allergic reactions to transfusion blood products per NHSN/CDC criteria^[[2]](#endnote-2)^
6. Known IgA deficiency
7. Females who are pregnant or breastfeeding.

*Use of remdesivir as treatment for COVID-19 is permitted.

**Table: Schedule of Events**

| Study period | Screen | Baseline | Infusion^[[3]](#footnote-1)^ | Follow-up | | | | |
| --- | --- | --- | --- | --- | --- | --- | --- | --- |
| Day | -2 to 0 | | 0 | 1±1 | 3 ± 1 | 7 ± 2 | 14 ± 2 | 28 ± 3 |
| Procedure |  |  |  |  |  |  |  |  |
| Informed consent | x |  |  |  |  |  |  |  |
| Demographic and Medical history | x |  |  |  |  |  |  |  |
| COVID-19 symptom screen | x |  |  |  |  |  |  |  |
| SARS-CoV-2 RT-PCR for eligibility^[[4]](#footnote-2)^ | x |  |  |  |  |  |  |  |
| Pregnancy test ^[[5]](#footnote-3)^ | x |  |  |  |  |  |  |  |
| ABO^[[6]](#footnote-4)^ | x |  |  |  |  |  |  |  |
| Randomization |  | x |  |  |  |  |  |  |
| Drug infusion^[[7]](#footnote-5)^ |  |  | x |  |  |  |  |  |
| Study Procedures | | | | | | | | |
| Vital signs | x | x | x^[[8]](#footnote-6)^ | x | x | x | x | x |
| Targeted physical examination | x |  | x | x | x | x | x | x |
| COVID-19 symptom screen | x | x | x | x | x | x | x | x |
| Concomitant medications | x | x | x |  |  |  |  |  |
| Assessment of composite outcome of disease severity^[[9]](#footnote-7)^* |  | x |  | x | x | x | x | x |
| Adverse event monitoring* |  |  | x | x | x | x | x | x |
| Blood taken for lab assessments: CBC and CMP* |  | x |  | x | x | x | x | x |
| Samples for future genetic testing^[[10]](#footnote-8)^ |  | x |  |  |  | x | x |  |
| SARS-CoV-2 RT-PCR^[[11]](#footnote-9)^ |  | x |  |  |  | x | x |  |

*Scheduled to be performed daily as per standard of care

### Subject Withdrawal

1. Subjects can terminate study participation and/or withdraw consent at any time without prejudice.
2. Randomized subjects who withdraw from the study will not be replaced.
3. The investigator may withdraw subjects if they are lost to follow up, non-compliant with study procedures or if the investigator determines that continued participation in the study would be harmful to the subject or the integrity of the study data.
4. Discontinuation of the study: The study sponsor, FDA and IRB all have the right to terminate this study at any time.

### Intervention

1. Subjects will be randomized in a 2:1 ratio to receive treatment vs. frozen fresh plasma.
2. Study drug: The investigational product is anti-SARS-CoV-2 plasma. Patients identified as having recovered from COVID-19 will serve as potential donors. Testing will confirm presence of anti-SARS-CoV-2 antibody prior to donation. Potential donors and samples will be screened for transfusion-transmitted infections (e.g. HIV, HBV, HCV, WNV, HTLV-I/II, *T. cruzi*, ZIKV) and plasma will be collected using apheresis technology. This is similar to standard blood bank protocols.
3. Active arm will receive 1 unit of anti-SARS-CoV-2 plasma.
4. Control arm will receive 1 unit of control plasma.
5. The study drug will be in a standard plasma unit bag, with a study-specific ISBT label and will include the following statement: “Caution: New Drug--Limited by Federal (or United States) law to investigational use."
6. The blood bank will not be blinded to treatment allocation, nor will the clinical study team.
7. The patient and the clinician who will assess the end of treatment outcome will be blinded to treatment allocation.

### Randomization

Subjects enrolled in the study will be randomized to receive study drug vs. control using a web-based randomization platform that will pre-generate all treatment assignments in a 2:1 ratio using random permuted blocks of random block sizes. The assignment list is maintained by designated staff at the DCC, independent from the study, and then sent to the Principal Investigator/research team for each participant that is deemed eligible.

## Study product considerations

The preparation of the anti-SARS plasma and the control plasma will take place at the New York City Blood Center and the CUIMC-NYPH Blood Bank will dispense the plasma products. The plasma collection procedures are not part of this research protocol and are described in separate protocols, which has separate IRB approval (AAAS9845). The description below provides a summary of study product considerations as context.

### Collection

All activities pertaining to the collection and processing of plasma will take place at [New York Blood Center/NYBC]. NYBC is one of the largest independent, community-based, nonprofit blood centers in the United States. NYBC has a longstanding research program and is well versed in the regulatory and ethical aspects of research, including clinical trials. The organization is FDA-licensed to produce convalescent plasma and AABB (American Association of Blood Banks) accredited, attesting to robust quality oversight of all operations.

The donation and collection of donor convalescent plasma will occur under CUIMC IRB protocol AAAS9845.

### Collection and processing

- Standard apheresis plasma collection will be performed per routine standard operating procedure at the collection facility (NYBC).
- As per routine practice, samples will be collected at time of donation for testing for transfusion-transmissible infections (all donors), ABO and red cell antibodies (all donors) and HLA antibodies (female donors with prior pregnancies).
- Target collection volume: ~450-600mL; this will allow for later splitting (separation) into 200-250mL daughter units.
- The plasma will be processed per routine practice; it will be frozen within 24hrs of collection per AABB standards.
- The plasma will be maintained in quarantine at the blood center pending laboratory test results (i.e. infectious screening, ABO and RhD status, Red cell and HLA antibodies).
- If laboratory testing is acceptable (i.e. negative infectious and antibody screening), the products will be distributed to hospital blood bank for storage.
- In the event of an abnormal test result, the product will be discarded and the donor will be notified by the blood center as is standard practice.

### 4.4.3. Control arm plasma

The control arm plasma follows identical collection and processing procedures, but will have been collected from community blood donors prior to documented SARS-CoV-2 in the United States (i.e., to be conservative all control arm plasma will be from collections prior to 31 December 2019).

### 4.4.4. Rationale for dosing

We will utilize 1 unit (200-250 mL) of plasma with anti-SARS-CoV-2 antibody.

Dosing was based on experience with previous use of convalescent plasma therapy in SARS1 where 5 mL/kg of plasma at titer ≥ 1:160 was utilized [European journal of clinical microbiology & infectious diseases: official publication of the European Society of Clinical Microbiology. 2005; 24(1):44-6.]. Historical precedence allowing for 0.25 of treatment dose was taken into account. Hence, considering first order linear proportionality, 3.125mL/kg of plasma with titer >1:64 would provide equivalent immunoglobulin level to one quarter of 5ml/kg plasma with titer ≥ 1:160. For a typical patient (~80 Kg) this would result in 250 mL of plasma (3.125ml/kg x 80kg = 250 mL > 1:64).

### 4.4.5. Study drug administration

- Drug will be administered within 24-48 hours of randomization.
- Infusion rate ≤ 250 mL/hour at physician discretion
- Pretreatment to minimize transfusion reactions (e.g. acetaminophen, diphenhydramine) will not be given, but will be available as needed to treat fever or allergic reaction. For severe allergic reactions corticosteroids (e.g., 125mg solu-medrol IV) may be used. For rare severe anaphylaxis, epinephrine will be available.
- If an AE develops during infusion, the infusion may be slowed or stopped as per investigator’s decision.
  - Most reactions to plasma are relatively minor and the infusion can generally be continued. Infusion site burning and non-allergic systemic effects can generally be managed with slowing of the infusion. Infusion is generally stopped in cases of itching; participant is treated and then infusion cautiously re-started.
  - Severe allergic reactions generally require discontinuation of the infusion. These include:
    - Respiratory compromise: dyspnea, wheezing, stridor, hypoxemia
    - A decrease in systolic blood pressure to < 90 mmHg or >30% decrease from baseline or a diastolic drop of >30% from baseline.
    - Tachycardia with an increase in resting heart rate to > 130bpm; or bradycardia <40 that is associated with dizziness, nausea or feeling faint.
    - Syncope
    - Confusion
    - Any other symptom or sign which in the good clinical judgment of the study clinician or supervising physician warrants halting the infusion. For example, the rapid onset of gastrointestinal symptoms, such as nausea, vomiting, diarrhea, and cramps, for instance, may be manifestations of anaphylaxis and may warrant an immediate halt prior to meeting full SAE criteria.

### Concomitant medications will be documented on the CRF

- Prescription medications
- Over the counter medications
- Herbal treatments/nutritional supplements
- Blood products

### Prohibited Medications:

Any approved or investigational drug* with established or potential activity against SARS-CoV-2 given within 24 hours of plasma infusion.

*Concurrent use of Remdesivir as treatment for COVID-19 during the course of the study is permitted.

# STATISTICAL CONSIDERATIONS AND DATA MANAGEMENT

## Design Overview

This randomized blinded phase 2 trial will assess the efficacy and safety of anti-SARS-CoV-2 plasma among adults with severe COVID-19. Eligible participants will be randomized in a 2:1 ratio to receive anti-SARS-CoV-2 plasma or fresh frozen plasma without known anti-SARS-CoV-2 antibodies. We plan to enroll a total of 129 participants (with 86 to plasma and 43 to standard of care). Each participant will be evaluated at baseline and daily during the follow-up period using a seven-category severity scale:

1. Not hospitalized with resumption of normal activities
2. Not hospitalized, but unable to resume normal activities
3. Hospitalized, not requiring supplemental oxygen
4. Hospitalized, requiring supplemental oxygen
5. Hospitalized, requiring high-­‐flow oxygen therapy or noninvasive mechanical ventilation
6. Hospitalized, requiring extracorporeal membrane oxygenation (ECMO), invasive mechanical ventilation, or both
7. Death

According to the inclusion/exclusion criteria, all enrolled participants will have a score ranging 3—6 on this scale at baseline. The primary study endpoint is defined as the time to achieve a one-point improvement on the scale.

Safety will also be evaluated daily. Secondary endpoints include

- SARS-CoV-2 PCR positivity from nasopharyngeal swab, collected on baseline (Day 0), Days 7, and 14.
- Levels of SARS-CoV-2 RNA on Days 0, 7, and 14.
- During of need for supplemental oxygen and/or mechanical ventilation
- Duration of hospitalization
- Mortality

## Statistical Analysis

- ***Efficacy Objective***

# **Primary Analysis, go/no-go decision, and power consideration**

The time to one-point-improvement from randomization will be compared in the framework of Cox proportional hazards model. Specifically, the hazards ratio for improvement will be tested using a Wald test: if the one-sided P-value favoring the plasma arm is less than 0.15, it will constitute a “go decision” in this proof-of-concept phase 2. The analysis will be intent-to-treat. Given the short study duration and the nature of the treatment, however, we anticipate minimal non-compliance or dropout.

With a 2:1 randomization ratio, under an assumed hazard ratio of 1.5, a one-sided Wald test at 0.15 level will require a total of 75 events of improvement in order to achieve 80% power. We consider a hazard ratio of 1.5 as minimally clinically relevant, and note that a recent study of antiviral drug yielded a hazard ratio of about 1.39. Given the plasma therapy, we expect a hazard ratio of 1.5 is realistic. Assuming that about 70% of the enrolled participants will achieve improvement, we determined a total of N = 129 (with 86 in plasma and 43 in SOC) will provide adequate power to achieve a go decision.

# **Secondary Analysis**

All secondary efficacy analyses will also be intent-to-treat.

Day-28 rating of the severity scale will be analyzed using Mann Whitney test. Longitudinal data collected over multiple days during the study period (e.g., PCR positivity, RNA) will be analyzed using the framework of generalized linear mixed model. Time-to-event variables (e.g., time to death) will be analyzed using Cox proportional hazards model. Continuous variables (e.g. duration of hospitalization) will be analyzed using Mann Whitney test. Treatment effects on these variables will be estimated with 95% confidence intervals.

# **Safety Analyses**

Serious adverse events will be summarized by grades and types using proportions and 95% confidence intervals for the two study arms. Relative safety profile of the two arms will be compared using Fisher’s exact test.

# **Missing data and non-compliance**

We will compare the missing data patterns between the study arms; and perform sensitivity analyses using different imputation approaches. However, due to the short study period, we anticipate minimal missing data. All efficacy analyses will be done intent-to-treat, although as-treat analyses will also be conducted as sensitivity.

## Go/no-go Decision and Power Consideration

The go/no-go decision will be based on a one-sided Wald test at 0.15 level. That is, a “go-decision” will be a one-sided P < 0.15, suggesting evidence of promise for further investigation in a Phase 3 trial. Adaptive seamless phase 2/3: The results in this proof-of-concept study will be used to plan the sample size in a Phase 3 trial, which may include the data in this Phase 2 in the final analysis using adjusted P value as in a seamless phase 2/3 trial. Details of P value adjustment and sample size calculation will be decided before the final analysis of this study data.

*Power Consideration*: With a 2:1 randomization ratio, under an assumed hazard ratio of 1.5, a one-sided Wald test at 0.15 level will require a total of 75 events of improvement in order to achieve 80% power. Assuming that about 70% of the enrolled participants will achieve improvement, we determined a total of N = 129 (with 86 in plasma and 43 in SOC) will provide adequate power to achieve a go decision.

# STUDY PROCEDURES

## Screening

1. Screening (must be completed before randomization)
2. Informed consent (obtained before performing study related activities)
3. Baseline Evaluation (at screening)
4. Demographics (age, sex, ethnicity, race)
5. Medical history (acute and chronic medical conditions, medications, allergies) (any medical condition arising after consent should be recorded as AE)
6. COVID-19 symptom screen (fever, cough, shortness of breath)
7. Confirmation of SARS-CoV-2 testing (RT-PCR) for eligibility (within 14 days of randomization)
8. Vital signs
9. Physical examination
10. Blood typing
11. Urine or serum pregnancy test for females of childbearing potential. Results from laboratory tests obtained up to 7 days before enrollment may be used for the pregnancy test.
12. Determination of eligibility as per inclusion/exclusion criteria

## Baseline (Randomization)

1. Randomization of eligible subject
2. Vital signs
3. COVID-19 symptom screen (fevers, cough, shortness of breath)
4. New medical conditions, concomitant medication
5. Assessment of clinical status (using 7-point ordinal outcome scale)
6. CBC, comprehensive metabolic panel (abstracted from routine clinical lab results in electronic medical record)
7. Stored samples for future studies (optional)
8. SARS-CoV-2 testing (RT-PCR) from nasopharyngeal swab (optional)

## Day 0 Infusion (Within 24-48 hours from randomization)

1. Study Plasma Administration: A single unit of plasma will be transfused. Time at start and end of infusion will be recorded and vital signs will be measured immediately prior to infusion, 10-20 minutes after start of infusion, and at completion of infusion.
2. Vital signs
3. Physical examination (acceptable to use clinician notes from electronic medical record)
4. COVID-19 symptom screen (fevers, cough, shortness of breath)
5. New medical conditions, concomitant medication, AE evaluation

## Day 1 ± 1

1. Vital signs
2. COVID-19 symptom screen (fevers, cough, shortness of breath)
3. Assessment of clinical status (using 7-point ordinal outcome scale)
4. New medical conditions, AE evaluation
5. Physical examination (acceptable to use clinician notes from electronic medical record)
6. CBC, comprehensive metabolic panel (abstracted from routine clinical lab results in electronic medical record)
7. Stored samples for future studies (optional)

## Day 3 ± 2

1. Vital signs
2. COVID-19 symptom screen (fevers, cough, shortness of breath)
3. Assessment of clinical status (using 7-point ordinal outcome scale)
4. New medical conditions, AE evaluation
5. Physical examination (acceptable to use clinician notes from electronic medical record)

## Day 7 ± 2

1. Vital signs
2. COVID-19 symptom screen (fevers, cough, shortness of breath)
3. Assessment of clinical status (using 7-point ordinal outcome scale)
4. New medical conditions, AE evaluation
5. Physical examination (acceptable to use clinician notes from electronic medical record)
6. SARS-CoV-2 testing (RT-PCR) from nasopharyngeal swab (optional)
7. CBC, comprehensive metabolic panel (abstracted from routine clinical lab results in electronic medical record)
8. Stored samples for future studies (optional)

## Day 14 ± 2

1. Vital signs
2. COVID-19 symptom screen (fevers, cough, shortness of breath)
3. Assessment of clinical status (using 7-point ordinal outcome scale)
4. New medical conditions, AE evaluation
5. Physical examination (acceptable to use clinician notes from electronic medical record)
6. SARS-CoV-2 testing (RT-PCR) from nasopharyngeal swab (optional)
7. CBC, comprehensive metabolic panel (abstracted from routine clinical lab results in electronic medical record)
8. Stored samples for future studies (optional)

## Day 28 ± 3

1. Vital signs
2. COVID-19 symptom screen (fevers, cough, shortness of breath)
3. Assessment of clinical status (using 7-point ordinal outcome scale)
4. New medical conditions, AE evaluation
5. Physical examination (acceptable to use clinician notes from electronic medical record)

# EFFICACY, VIROLOGIC AND PK MEASURES

**Primary Endpoint:** Time-to-clinical improvement, defined as the time from randomization to either an improvement of one point on a seven-category ordinal scale or alive discharge from the hospital, whichever comes first. The seven-category ordinal scale consists of the following categories:

1. Not hospitalized with resumption of normal activities
2. Not hospitalized, but unable to resume normal activities
3. Hospitalized, not requiring supplemental oxygen
4. Hospitalized, requiring supplemental oxygen
5. Hospitalized, requiring high-flow oxygen therapy or noninvasive mechanical ventilation
6. Hospitalized, requiring extracorporeal membrane oxygenation (ECMO), invasive mechanical ventilation, or both
7. Death

#### Virologic measures

1. Rates and duration of SARS-CoV-2 PCR positivity (RT PCR) at days 0, 7 and 14.
2. Peak quantity levels of SARS-CoV-2 RNA at days 0, 7 and 14.

# 8. RISKS AND BENEFITS

**8.1. Potential benefits of treatment**

The potential benefits of antiviral treatment with anti-SARS CoV-2 plasma in patients with severe COVID-19 is unknown. However, based on available evidence from use of convalescent plasma in SARS-CoV-1 patients, shortened duration of illness and improved mortality are potential benefits.

**8.2. Potential benefits of clinical monitoring and virologic testing**

Subjects enrolled in the study will undergo close virological monitoring that may facilitate improved understanding of viral shedding that may have benefit to the individual, their family and the community at large.

**8.3. Potential risks of study procedures**

1. Risks of plasma: Fever, chills, rash, headache, serious allergic reactions, transmission of infectious agents
2. Transfusion related acute lung injury (TRALI) and transfusion related circulatory overload (TACO), both of which may worsen oxygen saturation and increase work-of-breathing
3. Risks of phlebotomy: local discomfort, bruising, hematoma, bleeding, fainting,
4. Total blood draws will not exceed 500 mL
5. Risks of nasopharyngeal swab: local discomfort, vomiting
6. Risks of IV placement: bleeding, infection, thrombosis

**8.4. Potential risks of genetic testing**

Samples obtained for future research may include a search for genetic correlates of COVID-19 susceptibility or severity. Specimens will be labeled by study IDs, rather than names. This information will not be released to participants and will not become part of their medical records. Risks related to discrimination or other problems are deemed highly unlikely.

**8.5. Alternatives**

The alternative to participation in this study is continued standard-of-care clinical management.

**8.6. Safety monitoring**

1. Safety Evaluations: Will assess for the safety of anti-SARS-CoV-2 plasma in terms of treatment emergent adverse events.
2. Clinical evaluations: Vital signs and symptom screen on days 0-28 (or until hospital discharge, whichever is sooner).
3. Laboratory evaluations:
4. Safety laboratory tests (ABO typing, pregnancy testing, CBC and comprehensive metabolic panel) will be performed at the local CLIA-certified hospital clinical laboratory on days 0-14 (or until hospital discharge, whichever is sooner).

**8.7. Adverse event reporting**

An **Adverse Event (AE)**is any untoward or unfavorable medical occurrence in a human subject administered an investigational product, including any abnormal sign, symptom or disease, temporally associated with the subject’s participation in research, whether or not considered related to the subject’s participation in the research.

An AE does not include the following:

- Medical or surgical procedures such as surgery, endoscopy, tooth extraction, and transfusion. The condition that led to the procedure may be an AE and must be reported.
- Preexisting diseases, conditions, or laboratory abnormalities present or detected before the screening visit that do not worsen
- Situations where an untoward medical occurrence has not occurred (e.g., hospitalization for elective surgery, social and/or convenience admissions)
- Any medical condition or clinically significant laboratory abnormality with an onset date before the investigational product is administered to the subject and not related to a protocol-associated procedure is not an AE. It is considered to be preexisting and should be documented as medical history.

Preexisting events or conditions that increase in severity or change in nature after the subject receives the investigational product will be considered AEs.

**Serious Adverse Event (SAE)**:  any adverse event temporarily associated with the subject's participation in research that meets any of the following criteria:

- Results in death;
- Is life-threatening (places the subject at immediate risk of death from the event as it occurred);
- Requires inpatient hospitalization or prolongation of existing hospitalization;
- Results in a persistent or significant disability/incapacity;
- Results in a congenital anomaly/birth defect; or
- Any other adverse event that, based upon appropriate medical judgment, may jeopardize the subjects' health and may require medical or surgical intervention to prevent one of the other outcomes listed in this definition.

**Prolonged Hospitalization or Surgery**

Any AE that results in prolonged hospitalization should be documented and reported as a SAE. Any condition responsible for surgery should be documented as an AE if the condition meets the criteria for an AE.

Neither the condition, prolonged hospitalization nor surgery are reported as an AE in the following circumstances:

- Prolonged hospitalization for diagnostic or elective surgical procedures for a preexisting condition. Surgery should not be reported as an outcome of an AE if the purpose of the surgery was elective or diagnostic and the outcome was uneventful.
- Prolonged hospitalization for required to allow efficacy measurement for the study.

An **Unanticipated Problem (UP)**is any incident, experience or outcome involving risk to subjects or others in any human subjects research that meets all of the following criteria:

- Unexpected (in terms of nature, severity or frequency) given (a) the research procedures that are described in the IRB-approval protocol and informed consent document, and (b) the characteristics of the subject population being studied;
- Related or possibly related to participation in such research (i.e., there is a reasonable possibility that the incident, experience or outcome may have been caused by the procedures involved in such research); and
- Suggests that the research places subjects or others at a greater risk of harm (including physical, psychological, economic or social harm) than was previously known or recognized

A **Suspected Adverse Reaction (SAR)** is any AE for which there is a reasonable possibility that it was caused by the drug.

Reasonable possibility means that there is evidence to suggest a causal relationship between the drug and the AE.  Examples of reasonable possibility are:

- A single occurrence of an event that is uncommon and known to be strongly associated with drug exposure.
- One or more occurrences of an event that is not commonly associated with drug exposure, but is otherwise uncommon in the population exposed to the drug.
- An aggregate analysis of specific events observed in a clinical trial that indicates that those events occur more frequently in the drug treatment group than in a concurrent or historical control group.

**Investigator Reporting Requirements**

The Principal Investigator will report all AEs and SAEs to the IND sponsor within 48 hours of becoming aware of the event. The report to the IND sponsor will include the study investigator’s preliminary assessment of seriousness, severity and relatedness to the investigational product.

**To IRB:**

1. Unanticipated Problems (UPs) must be reported promptly, but not later than 7 calendar days following the occurrence of the UP or the Principal Investigator’s acquiring knowledge of the UP.

**To DSMB:**

Serious adverse events not constituting an unanticipated problem is to be reported to the DSMB and the IND sponsor. Reporting should occur within 48 hours of knowledge of the SAE occurrence.

**IND Sponsor Reporting Requirements**

The IND sponsor will report the following SARs to the FDA:

- To the FDA, as soon as possible, but no later than 7 calendar days after the S-I's initial receipt of the information, any **unexpected fatal or life-threatening SAR.**
- To the FDA and all participating investigators, as soon as possible but no later than 15 calendar days after the S-I determines that information qualifies for reporting, in an IND safety report, **any SAR that is both serious and unexpected.**
- To the FDA and all participating investigators, as soon as possible but no later than 15 calendar days after the S-I determines that the information qualifies for reporting, **any findings from epidemiological studies, pooled analysis of multiple studies or clinical studies, whether or not conducted under an IND or by the S-I, that suggest a significant risk in humans exposed to the drug**.
- To the FDA and all participating investigators, as soon as possible, but no later than 15 calendar days after the S-I determines that the information qualifies for reporting, **any findings from animal or *in vitro* testing, whether or not conducted by the S-I, that suggest a significant risk in humans exposed to the drug**.
- To the FDA and all participating investigators, as soon as possible, but no later than 15 calendar days after the S-I determines that the information qualifies for reporting, **any clinically important increase in the rate of a** **Serious SAR over that listed in the protocol or Investigator Brochure.**
- Expected SAEs and AEs should be included in the IND Annual Reports.

**Follow-up** information to a safety report will be submitted as soon as the relevant information is available.  However, if the results of a sponsor’s investigation show that an adverse drug experience not initially determined to be reportable are so reportable, the sponsor must report such experience as soon as possible, but no later than 15 calendar days after the determination is made.

**Reporting Interval**

All AEs and SAEs will be documented from the first administration of study product. All AEs and SAEs will be followed until resolution even if this extends beyond the study-reporting period. Resolution of an adverse event is defined as the return to pre-treatment status or stabilization of the condition with the expectation that it will remain chronic.

The investigator is not obligated to actively seek SAEs after the protocol-defined follow up period; however, if the investigator learns of any SAEs that occur after the protocol-defined follow-up period has concluded and the event is deemed relevant to the use of the investigational product, the investigator should promptly document and report the event to the IND sponsor.

**Investigator’s Assessment of Adverse Events**

The determination of seriousness, severity, and causality will be made by a study investigator who is qualified (licensed) or qualified subinvestigator to diagnose adverse event information, provide a medical evaluation of adverse events, and classify adverse events based upon medical judgment. This includes but is not limited to physicians, physician assistants, and nurse practitioners.

Laboratory abnormalities without clinical significance will not be recorded as AEs or SAEs. However, laboratory abnormalities (e.g., clinical chemistry, hematology) that require medical or surgical intervention or lead to an interruption, modification, or discontinuation of the investigational product must be reported as AE, as well as a SAE, if applicable. In addition, laboratory or other abnormal assessments (e.g., x-rays, vital signs, electrocardiogram) that are associated with signs and/or symptoms must be recorded as an AE or SAE if they meet the definition of an AE or SAE. If the laboratory abnormality is part of a syndrome, record the syndrome or diagnosis (e.g., anemia), not the laboratory result (i.e., decreased hemoglobin). The grading of the laboratory AEs will be based on the toxicity tables in <https://www.niaid.nih.gov/research/dmid-safety-reporting-pharmacovigilance>.

**Assessment of Seriousness**

Event seriousness will be determined according to the protocol definition of a Serious Adverse Event (SAE).

**Assessment of Severity**

Event severity will be assigned according to the Toxicity Tables. For parameters not included in the Toxicity Table the following definitions will be used:

1 = Mild: Transient or mild discomfort (<48 hours); no medical intervention/therapy required.)

2 = Moderate: Mild to moderate limitation in activity-some assistance may be needed; no or minimal medical intervention/therapy required)

3 = Severe: Marked limitation in activity, some assistance usually required; medical intervention/therapy required, hospitalizations possible

4 = Life-threatening: Extreme limitation in activity, some assistance usually required; medical intervention/therapy required, hospitalization or hospice care probable

5= Death

For AEs associated with laboratory abnormalities, the event should be graded on the basis of the clinical severity in the context of the underlying conditions; this may or may not be in agreement with the grading of the laboratory abnormality.

**Assessment of Expectedness**

Assessment of expectedness for SAEs will be determined by using safety information specified in the protocol, consent form or available information on the product, as applicable.

**Assessment of Causality**

The investigator or qualified subinvestigator is responsible for preliminary assessment of AEs and SAEs for the relationship to the investigational product using clinical judgement and the following considerations:

- No: Evidence exists that the AE has an etiology other than the investigational product. For SAEs, an alternative causality must be provided (e.g., preexisting condition, underlying disease, intercurrent illness, or concomitant medication).
- Yes: There is reasonable possibility that the AE may have been caused by the investigational product.

Relatedness will be documented on the level of certainty:

- Related
- Probably related
- Possibly related
- Unlikely
- Unrelated

The investigator must provide an assessment of association or relationship of AEs to the study product based on:

- Temporal relationship of the event to the administration of study product;
- Whether an alternative etiology has been identified;
- Biological plausibility;
- Existing therapy and/or concomitant medications.

# 9. SAFETY OVERSIGHT

## Monitoring Plan

1. All AEs and SAEs will be reviewed by the study team in real time.
2. A data safety monitoring board (DSMB), composed of independent experts without conflict of interests will be established. The Board will review the study before initiation and quarterly thereafter. The Board will review study data to evaluate the safety, efficacy, study progress, and conduct of the study.

## Study monitoring

As per ICH-GCP 5.18 and FDA 21 CFR 312.50, clinical protocols are required to be adequately monitored by the study sponsor. Monitors will verify that

- - - 1. There is documentation of the informed consent process and signed informed consent documents for each subject
      2. There is compliance with recording requirements for data points
      3. All SAEs are reported as required
      4. Individual subjects’ study records and source documents align
      5. Investigators are in compliance with the protocol.
      6. Regulatory requirements as per Office for Human Research Protections (OHRP), FDA, and applicable guidelines (ICH-GCP) are being followed.

## Halting Criteria for the Study

The study enrollment and dosing will be stopped and an ad hoc review will be performed if any of the specific following events occur or, if in the judgment of the study physician, subject safety is at risk of being compromised:

- - - 1. Unexpected death of a dosed subject in relation to infusion
      2. Occurrence of a life-threatening allergic/hypersensitivity reaction (anaphylaxis), manifested by bronchospasm with or without urticaria or angioedema requiring hemodynamic support with pressor medications or mechanical ventilation.
      3. One subject with an unexpected SAE associated with study product.
      4. Two subjects with a Grade 3 or higher toxicity for the same parameter associated with study product.
      5. An overall pattern of symptomatic, clinical, or laboratory events that the medical monitor, ISM, or SMC consider associated with study product and that may appear minor in terms of individual events but that collectively may represent a serious potential concern for safety.
      6. Any other event(s) which is considered to be a serious adverse event in the good clinical judgment of the responsible physician. This will be appropriately documented.

Furthermore, given that ADE may be an issue with convalescent antibody treatment, out of an abundance of caution we will monitor the number of subjects in each trial arm that progresses to an indication for need of mechanical ventilation. In monitoring the number of subjects that progresses to this stage, we will present these data to the DSMB and a masked outcomes accesfsor so that they may objectively evaluate and determine whether they would like to be unmasked. After at least 50% of trial participants have accumulated follow-up, the number of subjects that progress to this stage will be presented to the masked outcomes accessor and formally asked whether they (1) see a clinically meaningful difference between trial arms and (2) if so do they require a formal interim analysis. At any point should the DSMB require a formal interim analysis, we will examine the difference in treatment arms for need for mechanical ventilation. This interim analysis will adjust for factors related to need for mechanical ventilation including age and presence of cardiopulmonary comorbidities.

Upon completion of this review and receipt of the advice of the DSMB, the IND sponsor will determine if study entry or study dosing should be interrupted or if study entry and study dosing may continue according to the protocol. Should the trial not be stopped at this time point, the final analysis would need to account the number of interim analyses that were conducted. Therefore, we would penalize any final analysis dividing our 0.05 alpha in half for each interim analysis.

**Halting Criteria/Rules for Subject Infusion**

Infusion of study drug will be halted if any of the following manifestations of anaphylaxis develop and will not be restarted:

- Skin or mucous membrane manifestations: hives, pruritus, flushing, swollen lips, tongue or uvula
- Respiratory compromise: dyspnea, wheezing, stridor, hypoxemia
- A decrease in systolic blood pressure to < 90 mmHg or >30% decrease from baseline or a diastolic drop of >30% from baseline.
- Tachycardia with an increase in resting heart rate to > 130bpm; or bradycardia <40 that is associated with dizziness, nausea or feeling faint.
- Syncope
- Confusion
- Any other symptom or sign which in the good clinical judgment of the study clinician or supervising physician warrants halting the infusion. For example, the rapid onset of gastrointestinal symptoms, such as nausea, vomiting, diarrhea, and cramps, for instance, may be manifestations of anaphylaxis and may warrant an immediate halt prior to meeting full SAE criteria

# ETHICS/PROTECTION OF HUMAN SUBJECTS

## Ethical Standard

The investigators are committed to the integrity and quality of the clinical studies it coordinates and implements. The investigators will ensure that the legal and ethical obligations associated with the conduct of clinical research involving human subjects are met. The information provided in this section relates to all sites participating in this research study.

As the Department of Health and Human Services continues to strengthen procedures for human subjects’ protections via new regulations, the investigators will review these evolving standards in relation to the proposed activities and will advise the investigators on those that may apply.

In addition, The Trustees of Columbia University in the City of New York, has a Federalwide Assurance (FWA) number on file with the Office for Human Research Protections (OHRP). The FWA number for CU is FWA00002636.

This assurance commits a research facility to conduct all human subjects’ research in accordance with the ethical principles in The Belmont Report and any other ethical standards recognized by OHRP. Finally, per OHRP regulations, the research facility will ensure that the mandatory renewal of this assurance occurs at the times specified in the regulations.

## Institutional Review Board

The Columbia University Irving Medical Center (CUIMC) IRB will review this protocol and all protocol-related documents and procedures as required by OHRP and local requirements before subject enrollment. The CUIMC IRB currently holds and will maintain a US FWA issued by OHRP for the entirety of this study.

## Informed Consent Process

The informed consent process will be initiated before a volunteer agrees to participate in the study and should continue throughout the individual’s study participation. The subject or their legally authorized representative (LAR) will sign the informed consent document before any procedures are undertaken for the study. A copy of the signed informed consent document will be given to the subject and/or their representative for their records. The consent will explain that subjects may withdraw consent at any time throughout the course of the trial. Extensive explanation and discussion of risks and possible benefits of this investigation will be provided to the subjects and/or their representatives in understandable language. Adequate time will be provided to ensure that the subject has time to consider and discuss participation in the protocol.

The consent will describe in detail the study interventions/products/procedures and risks/benefits associated with participation in the study. The rights and welfare of the subjects will be protected by emphasizing that their access to and the quality of medical care will not be adversely affected if they decline to participate in this study.

## Subject Confidentiality

Subject confidentiality is strictly held in trust by the participating investigators, their staff, and the sponsors and their agents. No information concerning the study or the data will be released to any unauthorized third party without prior written approval of the sponsor. The results of the research study may be published, but subjects’ names or identifiers will not be revealed. Records will remain confidential. To maintain confidentiality, the PI will be responsible for keeping records in a locked area and results of tests coded to prevent association with subjects’ names. Data entered into computerized files will be accessible only by authorized personnel directly involved with the study and will be coded. Subjects’ records will be available to the FDA, the NIH, the manufacturer of the study product and their representatives, investigators at the site involved with the study, and the IRB.

## Future Use of Stored Specimens

Subjects or their LAR will be asked for consent to use their samples for future testing before the sample is obtained. The confidentiality of the subject will be maintained. They will be no plans to re-contact them for consent or to inform them of results. The risk of collection of the sample will be the small risk of bruising or fainting associated with phlebotomy however these samples will be taken at the same time as other protocol required samples.

DNA and RNA analyses of host cells may be done to identify factors that can improve diagnosis and treatment of COVID. Five ml of blood samples will be collected at 5 time points (See Schedule of Events). Serum will be frozen in 1-ml aliquots. These samples will be used to answer questions that may arise while the study is underway or after it is completed. If for instance, there were unanticipated AEs, serum could be used to run tests that might help determine the reason for the AEs. Cytokines could be measured, for example.

Samples would not be shared with investigators other than investigators at CUIMC unless outside investigators had relevant assays or expertise not available to the study investigators. The specimens would remain linked and at CUIMC for 5 years. Any use of these specimens not specified in the current protocol will be reviewed by the CUIMC IRB.

## Data management and monitoring

### Source Documents

Source documents may include research records documenting study procedures, laboratory test reports, and any medical records that may be generated during the study period (e.g. hospitalization due to adverse events or disease progression). These will be considered the source documents for the purposes of auditing the study. The investigator will retain a copy of source documents. The investigator will permit monitoring and auditing of these data, and will allow the sponsor, IRB and regulatory authorities access to the original source documents. The investigator is responsible for ensuring that the data collected are complete, accurate, and recorded in a timely manner. Source documentation (the point of initial recording of information) should support the data collected and entered into the study database/case report form and must be signed and dated by the person recording and/or reviewing the data. All data submitted should be reviewed by the site investigator and signed as required with written or electronic signature, as appropriate. Data entered into the study database will be collected directly from subjects during study visits or will be abstracted from subjects’ medical records. The subjects’ medical records must record their participation in the clinical trial and what medications (with doses and frequency) or other medical interventions or treatments were administered, as well as any AEs experienced during the trial.

### Data Management Plan

All paper documents will be maintained under double-locked conditions and will be stored separately from all other study records. Similarly, any electronically captured data will be stored in a unique database on a secure server separate from study databases. Only authorized study staff will have access to forms and databases with study data.

The study database will be programmed using REDCap, which is 21 CFR Part 11 and HIPAA compliant, and hosted in an Amazon Web Services (AWS). AWS has a Business Associates Agreement (BAA) with Columbia University. Study data will be collected at the study site(s) and entered into the study database. Data entry is to be completed on an ongoing basis during the study.

All study data will be collected during study visits using encrypted and password-protected tablets, laptops or computers.

The database will include automated quality checks that flag outliers, suspicious distributions and means, values that violate frequency or numeric thresholds, and error-prone submissions.

User permissions will be adjusted according to staff role and responsibility. Only designated study staff will access study data from the encrypted server to conduct data cleaning and quality checks, and generate reports to monitor study progress. Only authorized staff will access PII data for the purpose of conducting study operations. Study data will be collected at the study site(s) and entered into the study database or case report forms. Data entry is to be completed on an ongoing basis during the study.

### Study Record Retention

The site investigator is responsible for retaining all essential documents listed in the ICH GCP Guidelines. The FDA requires study records to be retained for up to 2 years after marketing approval or disapproval (21 CFR 312.62), or until at least 2 years have elapsed since the formal discontinuation of clinical development of the investigational agent for a specific indication. These records are also to be maintained in compliance with IRB/IEC, state, and federal medical records retention requirements, whichever is longest. All stored records are to be kept confidential to the extent provided by federal, state, and local law. It is the site investigator’s responsibility to retain copies of source documents until receipt of written notification to the sponsor.

No study document should be destroyed without prior written agreement between the sponsor and the Principal Investigator. Should the investigator wish to assign the study records to another party and/or move them to another location, the site investigator must provide written notification of such intent to sponsor with the name of the person who will accept responsibility for the transferred records and/or their new location. The sponsor must be notified in writing and written permission must be received by the site prior to destruction or relocation of research records.

# 11. REFERENCES

Arabi, Y. M., Hajeer, A. H., Luke, T., Raviprakash, K., Balkhy, H., Johani, S., . . . Alahmadi, M. (2016). Feasibility of Using Convalescent Plasma Immunotherapy for MERS-CoV Infection, Saudi Arabia. *Emerg Infect Dis, 22*(9), 1554-1561. doi:10.3201/eid2209.151164

Austin, P. C., & Fine, J. P. (2017). Practical recommendations for reporting Fine-Gray model analyses for competing risk data. *Stat Med, 36*(27), 4391-4400. doi:10.1002/sim.7501

Beigel JH, Tebas P, Elie-Turenne MC, Bajwa E, Bell TE, Cairns CB, et al. Immune plasma for the treatment of severe influenza: an open-label, multicentre, phase 2 randomised study. Lancet Respir Med. 2017; 5: 500-511

Cao B, Wang Y, Wen D, Liu W, Wang J, Fan G, et al. A Trial of Lopinavir-Ritonavir in Adults Hospitalized with Severe Covid-19. N Engl J Med. 2020; doi: 10.1056/NEJMoa2001282

Casadevall, A., & Pirofski, L. A. (2003). Antibody-mediated regulation of cellular immunity and the inflammatory response. *Trends Immunol, 24*(9), 474-478. doi:10.1016/s1471-4906(03)00228-x

Casadevall, A., & Scharff, M. D. (1994). Serum therapy revisited: animal models of infection and development of passive antibody therapy. *Antimicrob Agents Chemother, 38*(8), 1695-1702. doi:10.1128/aac.38.8.1695

Cheng, Y., Wong, R., Soo, Y. O., Wong, W. S., Lee, C. K., Ng, M. H., . . . Cheng, G. (2005). Use of convalescent plasma therapy in SARS patients in Hong Kong. *Eur J Clin Microbiol Infect Dis, 24*(1), 44-46. doi:10.1007/s10096-004-1271-9

Crowe, J. E., Jr., Firestone, C. Y., & Murphy, B. R. (2001). Passively acquired antibodies suppress humoral but not cell-mediated immunity in mice immunized with live attenuated respiratory syncytial virus vaccines. *J Immunol, 167*(7), 3910-3918. doi:10.4049/jimmunol.167.7.3910

Fine, J. P., Gray, R. J. (1999). A Proportional Hazards Model for the Subdistribution of a Competing Risk. *Journal of the Americal Statistical Association, 94*(446), 496-509. doi:10.1080/01621459.1999.10474144

Gunn, B. M., Yu, W. H., Karim, M. M., Brannan, J. M., Herbert, A. S., Wec, A. Z., . . . Alter, G. (2018). A Role for Fc Function in Therapeutic Monoclonal Antibody-Mediated Protection against Ebola Virus. *Cell Host Microbe, 24*(2), 221-233 e225. doi:10.1016/j.chom.2018.07.009

Kahan, B. C., Jairath, V., Dore, C. J., & Morris, T. P. (2014). The risks and rewards of covariate adjustment in randomized trials: an assessment of 12 outcomes from 8 studies. *Trials, 15*, 139. doi:10.1186/1745-6215-15-139

Ko, J. H., Seok, H., Cho, S. Y., Ha, Y. E., Baek, J. Y., Kim, S. H., . . . Peck, K. R. (2018). Challenges of convalescent plasma infusion therapy in Middle East respiratory coronavirus infection: a single centre experience. *Antivir Ther, 23*(7), 617-622. doi:10.3851/IMP3243

Liang, K., Zeger, S. L. (1986). Longitudinal data analysis using generalized linear models. *Biometrika, 73*(1), 13-22.

Mair-Jenkins, J., Saavedra-Campos, M., Baillie, J. K., Cleary, P., Khaw, F. M., Lim, W. S., . . . Convalescent Plasma Study, G. (2015). The effectiveness of convalescent plasma and hyperimmune immunoglobulin for the treatment of severe acute respiratory infections of viral etiology: a systematic review and exploratory meta-analysis. *J Infect Dis, 211*(1), 80-90. doi:10.1093/infdis/jiu396

Sahr, F., Ansumana, R., Massaquoi, T. A., Idriss, B. R., Sesay, F. R., Lamin, J. M., . . . Gevao, S. M. (2017). Evaluation of convalescent whole blood for treating Ebola Virus Disease in Freetown, Sierra Leone. *J Infect, 74*(3), 302-309. doi:10.1016/j.jinf.2016.11.009

van Erp, E. A., Luytjes, W., Ferwerda, G., & van Kasteren, P. B. (2019). Fc-Mediated Antibody Effector Functions During Respiratory Syncytial Virus Infection and Disease. *Front Immunol, 10*, 548. doi:10.3389/fimmu.2019.00548

Wan, Y., Shang, J., Sun, S., Tai, W., Chen, J., Geng, Q., . . . Li, F. (2020). Molecular Mechanism for Antibody-Dependent Enhancement of Coronavirus Entry. *J Virol, 94*(5). doi:10.1128/JVI.02015-19

Wang Y, Fan G, Salam A, Horby P, Hayden FG, Chen C, et al. Comparative Effectiveness of Combined Favipiravir and Oseltamivir Therapy Versus Oseltamivir Monotherapy in Critically Ill Patients With Influenza Virus Infection. J Infect Dis. 2020;221:1688-1698.

World Health Organization. Research and development blueprint: novel coronavirus. Geneva: World Health Organization, 2020. https://www.who.int/blueprint/priority-diseases/key-action/COVID19_Treatment_Trial_ Design_Master_Protocol _synopsis_Final_1 502 8022020.pdf. Accessed April 11th, 2020.

Xinhua. (2020). China puts 245 COVID-19 patients on convalescent plasma therapy. Retrieved from http://www.xinhuanet.com/english/2020-02/28/c_138828177.htm

Yeh, K. M., Chiueh, T. S., Siu, L. K., Lin, J. C., Chan, P. K., Peng, M. Y., . . . Chang, F. Y. (2005). Experience of using convalescent plasma for severe acute respiratory syndrome among healthcare workers in a Taiwan hospital. *J Antimicrob Chemother, 56*(5), 919-922. doi:10.1093/jac/dki346

Zhang, J. S., Chen, J. T., Liu, Y. X., Zhang, Z. S., Gao, H., Liu, Y., . . . Yin, W. D. (2005). A serological survey on neutralizing antibody titer of SARS convalescent sera. *J Med Virol, 77*(2), 147-150. doi:10.1002/jmv.20431

1. NHSN Biovigilance Component Hemovigilance Module Surveillance Protocol v2.5.. Atlanta, GA, USA: Centers for Disease Control and Prevention, 2018. [↑](#endnote-ref-1)
2. [↑](#endnote-ref-2)
3. Infusion will occur within 24-48 hours from Baseline (randomization). [↑](#footnote-ref-1)
4. Confirmation of +PCR from nasopharyngeal swab or oropharyngeal swab/tracheal aspirate samples within 14 days of randomization. [↑](#footnote-ref-2)
5. Urine pregnancy test for women of childbearing potential [↑](#footnote-ref-3)
6. Assessment of ABO type on file [↑](#footnote-ref-4)
7. Only participants randomized to the investigational drug will receive drug infusion [↑](#footnote-ref-5)
8. Vital sign testing: Immediately prior to infusion, 10-20 minutes after start of infusion, and at completion of infusion [↑](#footnote-ref-6)
9. Using 7-point ordinal scale [↑](#footnote-ref-7)
10. Additional blood for genetic assessments (optional) will be taken at a maximum of three of the indicated visits. [↑](#footnote-ref-8)
11. The nasopharyngeal swab for the PCR test (optional) will be collected up to four of the indicated time points. [↑](#footnote-ref-9)
